# Facile conversion of *cis* into *trans* oxane as liquid crystals

**DOI:** 10.1038/s41598-020-63902-y

**Published:** 2020-04-24

**Authors:** Zhaoheng Dong, Ping Fan, Qun Chen, Chen Chen

**Affiliations:** 1 Shandong Shenghua New Material Technology Co., Ltd., Laiyang city, 265110 P.R. China; 2 Nephrology Department, Shaanxi Province Hospital of Traditional Chinese Medicine, Xi’an city, 710003 P.R. China; 3 Institute of Endemic Diseases, Xi’an Jiaotong University Health Science Center, Xi’an, 710061 P.R. China; 4 School of Biomedical Sciences, University of Queensland, Queensland, 4072 Australia

**Keywords:** Catalyst synthesis, Liquid crystals

## Abstract

*Trans*-oxanes are important liquid crystals. The commonly used techniques for the synthesis were to react 2-substituted propylene glycols with substituted formaldehydes. Such process produces toxic *cis*-oxanes, which are harmful to the environment. The *cis* to *trans* isomerization of wasted *cis*-oxanes was studied in the presence of p-toluenesulfonic acid as catalyst. The yield of *cis* to *trans* conversion was over 70%, which was much higher than 42–69% when traditional methods were employed. The total yield of the new method was increased to 90%. Further investigation of effects of catalysts, reaction times, temperatures on the *cis-trans* conversion was carried out. Proposed mechanism of this process for the conversion was discussed.

## Introduction

**Figure 1:**
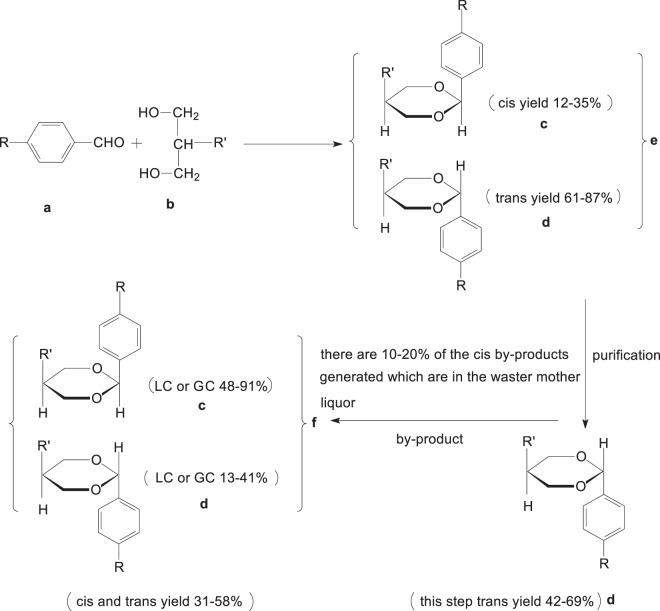
The commonly-used synthesis of pure *trans*-oxane (d). R: C_1–5_ linear alkyl, p-alkylphenyl or p-alkylphenyl cyclohexane; R’: p-ethylphenyl or p-cyanophenyl.

One feature of *trans*-oxane materials is easy to synthesize, which is a key requirement for the applications in TN/STN and TFT modes of liquid crystal display^1,2^. The class of *trans* oxane is among the key materials for liquid crystal display. During the past decades, the research on oxane liquid crystals, especially *trans*-oxanes, has attracted increasing attention in the field^3,4^. The commonly used synthesis was to react substituted formaldehydes (**a**) with 2-substituted propylene glycols (**b**) for mixed *cis*-*trans* oxanes (**e**)^5,6^, followed by separation and purification to give pure *trans*-oxane monomer (**d**) **(**Fig. 1).

Liquid crystal display requires highly pure *trans*-oxanes, and its *cis*-isomers have to be removed from their mixtures. The routine purification was to extract *trans*-isomers by recrystallization. However, such strategy was unsatisfactory^7,8^. Some amount of *trans*-isomers remained in the mother liquor with *cis*-isomers, therefore the yield of *trans*-oxanes was relatively low (about 50% as calculated based on alkyl benzaldehyde). After recrystallization a large amount of mother liquor (**f**) was left over containing mainly *cis-trans* mixtures. There is no literature on recycling the mixtures and purifying the *trans* isomers, so mixture **f** was usually discarded causing not only environmental pollution, but also high cost of the *trans* isomers.

**Figure 2:**
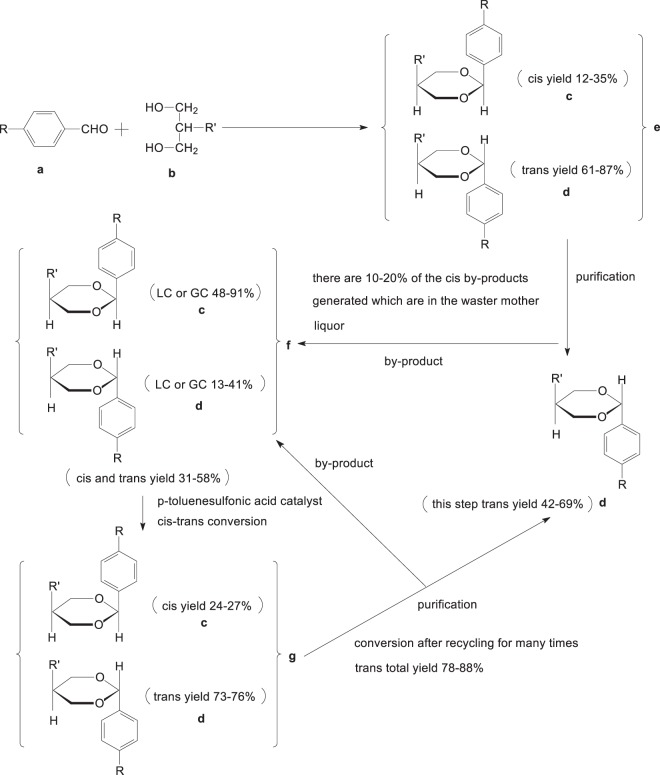
Conversion of *cis* into *trans* oxanes. R: C_1–5_ linear alkyl, p-alkylphenyl or p-alkylphenyl cyclohexane; R’: p-ethylphenyl or p-cyanophenyl.

Although *cis*-*trans* isomerization was reported^9,10^, there has been no reports on converting *cis*-oxanes (**c**) into *trans*-oxanes so far. In order to improve the yield of *trans*-oxanes and reduce the environmental pollution, a new synthetic route (Fig. 2) was employed to give *trans*-oxanes (**d**). As shown in Fig. 2, the *cis*-*trans* mixtures of oxanes (**e**) were synthesized by docking p-alkyl benzaldehyde (**a**) directly with 2-substituted propylene glycol (**b**). Pure *trans*-oxanes (**d**) monomers were obtained by either high vacuum distillation, chromatography or recrystallization. The latter left a large amount of *cis*-*trans* mixtures (**f**) with *cis* configuration dominant. The *cis*-*trans* mixture with *trans* configuration dominant (**g**) was obtained in the presence of p-toluenesulfonic acid as catalyst. Then the mixture (**g**) was purified further as shown in Fig. 2 to give pure *trans*-configuration of oxanes (**d**). The process was repeated several times until *cis* isomers were completely converted into *trans* ones.

Obviously the overall yield of *trans*-oxanes in Fig. 2 reached up to 78–88% (calculated based on alkyl benzaldehyde), which was significantly higher than that in Fig. 1 (about 42–69%). Due to the repeated conversion and purification of the mixture **f**, the mother liquor generated in the process was reused in site repeatedly to reduce environmental pollution. This improves the utilization rate of the starting materials and reduces the cost of products. Since the syntheses of corresponding compounds **a** and **b** were previously reported^11,12^, this paper will only deal with how to carry out the catalytic conversion of the key *cis* to *trans* isomers. The synthesis of representative mixture **e** was also selected to describe the conversion of *cis*-*trans* mixture **f**. The factors and mechanism of conversion were analyzed throughout the experiments in order to provide an example for the optimization of the process, and a reference for the synthesis of similar oxanes.

## Results

### The effect of the reaction conditions on the conversion

#### Reaction temperature

The effects of the reaction temperature on the *cis* to *trans* oxane conversion of mother liquors (**f**) were shown in Table 1. The temperatures of *cis* to *trans* isomerization of 2-p-cyanophenyl-5-ethyl-1,3-dioxane and 5-(4-n-propyl) cyclohexyl-2-p-ethylphenyl-1,3-dioxane were around 98 °C (n-heptane as solvent) to 110 °C (toluene as solvent). The corresponding *trans* isomers were obtained by recrystallization and the ratio of *cis*/*trans* conversion maintained at 25:75 as temperatures were increased.

**Table 1:** The effect of reaction temperature on conversion.

compound	The structure of the target compound	Reaction temperature/°C	The ratio of *cis-trans* before conversion	The ratio of *cis-trans* after conversion
**d1**		98	82:18	24:76
		110	82:18	25:75
**d2**		98	74:26	25:75
		110	74:26	25:75
**d3**		98	63:37	25:75
		110	63:37	25:75
**d4**		98	66:34	25:75
		110	66:34	25:75
**d5**		98	78:22	25:75
		110	78:22	25:75
**d6**		98	51:49	25:75
		110	51:49	24:76

#### The influence of reaction time

The influence of reaction time onthe *cis* to *trans* oxane conversion of mother liquors (**f**) was shown in Table 2. The *cis-trans* isomerization was completed in 1 h at 98 °C or 110 °C and there was no change of the *cis-trans* ratio when reaction time was increased. The proportion of *cis-trans* product remained at the level of 25:75 even though prolonged reaction time was applied.

**Table 2:** The effect of reaction time on conversion.

compound	The structure of the target compound	Reaction temperature/°C	The ratio of *cis-trans* after conversion at different reaction time			
			0 h	1 h	2 h	3 h
**d1**		98	82:18	24:76	25:75	25:75
		110	82:18	25:75	25:75	25:75
**d2**		98	74:26	25:75	25:75	25:75
		110	74:26	25:75	25:75	25:75
**d3**		98	63:37	25:75	25:75	25:75
		110	63:37	25:75	25:75	25:75
**d4**		98	66:34	25:75	25:75	25:75
		110	66:34	25:75	25:75	25:75
**d5**		98	78:22	25:75	25:75	25:75
		110	78:22	25:75	25:75	25:75
**d6**		98	51:49	25:75	25:75	25:75
		110	51:49	24:76	25:75	25:75

## Discussion

**Figure 3:**
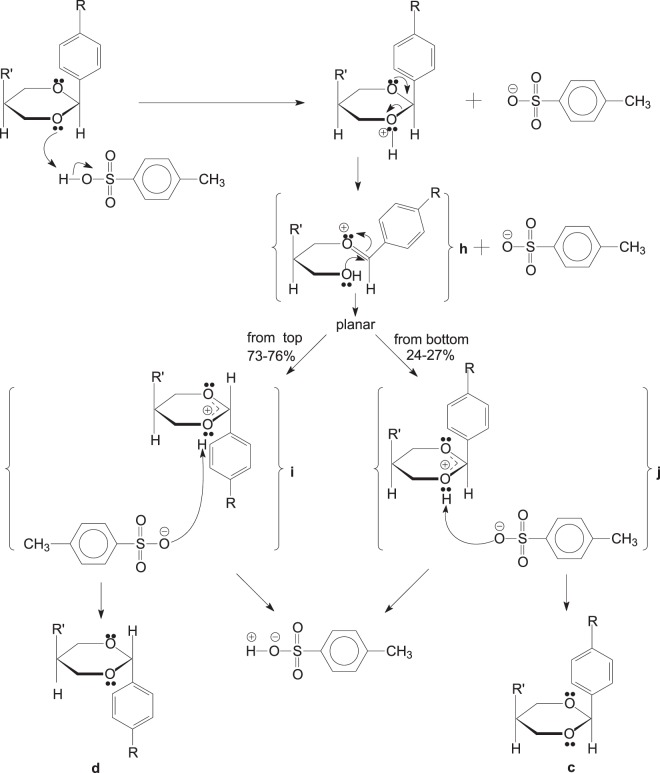
The proposed mechanism of the conversion.

It was known that the conversion of the *cis-trans* isomer might be a chemical dynamic equilibrium process^13^, which was generally divided into three categories: photoisomerzation, thermoisomerization and catalytic isomerization. As the final *cis-trans* equilibrium was decided by the thermodynamic stability of each isomer, thermoisomerization and catalytic isomerization were classified into one group during the synthesis^14^. The isomerization was mainly focused on C=C, C=N^15^. The studies on the catalytic isomerization of cycloalkanes and heterocycles^16^ were mainly concentrated on how to control *cis*-generation in ring-closure. However, the mechanism of how *cis*-isomers are converted into *trans*-ones is still unknown. It is also difficult to study the mechanism of catalytic isomerization because of the electronic and spatial effects of substituents on isomer molecules. Currently, only CNDO/2, SCF-MO and FSGO are used to roughly determine whether the mechanism of catalytic isomerization is based on the plane lateral displacement mechanism or the torsion mechanism under acid-base catalysis. We propose that the conversion process and the mechanism of the *cis-trans* isomerization of oxanes are as follows (Fig. 3):

Oxanes undergo ring opening in the presence of p-toluenesulfonic acid to go through a planar intermediate state **h**, followed by the aldol condensation for ring closure to give *cis* (**c**) an d *trans* (**d**) acetal, respectively. The rearrangement process favors *trans* configuration as it is more stable thermodynamically. The experimental results showed the ratio of *cis-trans* compounds in the system was 25:75, and it was basically unchanged (Tables 1 and 2). This means experimental results supported the proposed mechanism.

## Methods

### Materials and equipments

SY 25000-type high-pressure liquid chromatograph (HPLC, methanol as mobile phase, C218 as the stationary phase, a flow rate of 1 mL·min-1); 1102 gas chromatograph (GC); HP 5989B mass spectrometer analyzer. p-cyanobenzaldehyde, toluene, calcium chloride and 2-ethyl propylene glycol were from Shanghai Reagent Factory (analytical grade). The anhydrous magnesium sulfate was also analytical grade from Tianjin Sitong Chemical Plant.

### Preparation of the *cis-trans* mixture (e1) of 2-p-cyanophenyl-5-ethyl-1,3-dioxane

Toluene 130 mL, 2-ethylpropylene glycol 26.5 g (255 mM), p-cyanobenzaldehyde 32.8 g (250 mM), calcium chloride 0.5 g were successively added into the 500 mL reaction flask equipped with mechanical stirrer and reflux condenser. They were then stirred, and heated to reflux. Heating stopped when no water was generated. Analysis of the sample was performed on the gas chromatography (GC). When GC online results showed the content of p-cyanobenzaldehyde was less than 0.05%, the reaction was quenched. The mixture was cooled with water 150 ml. The aqueous phase was extracted with toluene 20 mL. The organic phases were combined and washed with water to neutral, then dried with anhydrous magnesium sulfate 5 g for 8 hrs and filtered. The filter cake was washed with toluene 10 mL twice. After distillation, the *cis-trans* mixture (**e1**) 52.2 g of 2-p-cyanophenyl-5-ethyl-1,3-dioxane was obtained. GC analysis showed that the content of the *cis*-2-p-cyanophenyl-5-ethyl-1,3-dioxane (**c1**) was ≥20% and the *trans* -2-p-cyanophenyl-5-ethyl-1,3-dioxane (**d1**) was ≥79%, respectively.

### How to prepare the *trans* -2-p-cyanophenyl-5-ethyl-1,3-dioxane

The **e1** prepared from above was recrystallized from ethanol at the ratio of 1 g/1.8 mL twice. The product was 34.6 g, and the purity of **d1** was ≥99.5%, analyzed by gas chromatography (GC) and the purified yield was about 65%. The *cis-trans* mixture containing 2-p-cyanophenyl-5-ethyl-1,3-dioxane (**f1**) 17.4 g was obtained from the combined recrystallization mother liquor and the solvent was removed by distillation. Analysis of the sample by GC showed that the content of **c1** was ≥58%, and **d1** was ≥39%. **f1** was directly used in following reaction.

### Conversion of c1 to d1 under catalystic isomerization in the presence of p-toluenesulfonic acid

The *cis-trans* mixture (**f1**) 17.4 g (82 mM), p-toluenesulfonic acid 1.7 g (10 mM) and toluene 100 mL were successively added into 250 mL reaction flask equipped with mechanical stirrer, reflux condenser. The reaction mixture was heated under stirring. Analysis of the sample was carried out every 0.5 h and reflux continued for 3 h. When GC online results showed the *cis-trans* ratio remained unchanged, the reaction was quenched. The mixture was cooled and water 50 mL was added. The aqueous phase was extracted with toluene 20 mL. The organic phases were combined, washed with water to neutral, dried over anhydrous magnesium sulfate 5 g for 8 hrs and filtered. The filter cake was washed with toluene 10 mL twice. The *cis-trans* mixture (**g1**) 17.4 g was obtained after solvent removal and distillation.

Analysis of the sample by GC showed that **c1** was ≥21%, and **d1** was ≥78%. After recrystallization for purification, the product (**d1**) was 12.1 g with purity ≥99.5% analyzed by GC, and the yield was ≥69%. The mother liquor from recrystallization (**f1**) was collected and extracted. The overall yield of compound **d1** based on p-cyanobenzaldehyde was ≥93% following the procedure of Fig. 2.

### Conversion of *cis*-5-(4-n-propyl) cyclohexyl-2-p-ethylphenyl-1,3-dioxane (c6) to its corresponding *trans* configuration (d6) in the presence of p-toluenesulfonic acid

The *cis-trans* mixture (**f6**) 23 g containing 5-(4-propyl) cyclohexyl-2-p-ethylphenyl-1,3-dioxane was obtained following the same experimental procedure as above. Analysis of the sample by GC showed that **c6** was ≥87% and **d6** was ≥12%. In 250 mL reaction flask equipped with mechanical stirrer, reflux condenser and addition funnel, 23 g (73 mM) of *cis-trans* mixture of **f6**, 1.1 g (7 mM) of p-toluenesulfonic acid and 100 mL of n-heptane were added to the reaction flask. Then reaction mixture was heated under stirring. Analysis of the sample was carried out every 0.5 h and reflux continued for 3 hrs. When GC online results showed the *cis-trans* ratio was unchanged, the reaction was quenched. Then the reaction mixture was cooled and water 50 mL was added. The aqueous phase was extracted with n-heptane 20 mL. The organic phases were combined and washed with water to neutral, dried over anhydrous magnesium sulfate 5 g for 8 hrs and filtered, then washed with n-heptane 10 mL twice. The *cis-trans* mixture (**g6**) was obtained after distillation. Analysis of the sample by GC showed that *cis*-5-(4-n-propyl) cyclohexyl-2-p-ethylphenyl-1, 3-dioxane (**c6**) ≥23%, and *trans*-5-(4-n-propyl) cyclohexyl-2-p-ethylphenyl-1, 3-dioxane (**d6**) was ≥76%. After recrystallization and purification, the product (**d6**) was 14.8 g, the purity of **d6** analyzed by GC was ≥99.5%, and the yield was ≥65%. The mother liquor from recrystallization was collected and extracted with the method described in Fig. 2. The overall yield of **d6** was ≥70%.
